# Biomedical Potential of the Deep-Sea Vent Mussel *Bathymodiolus azoricus*: Integrating Immunity, Bioadhesion, Biomineralization, and Targeted Transcriptomic Reanalysis

**DOI:** 10.3390/md24090318

**Published:** 2026-09-10

**Authors:** Raul Bettencourt, Rui L. Reis, Tiago H. Silva

**Affiliations:** 1 OKEANOS Institute, University of the Azores, 9901-862 Horta, Portugal; 2 Faculty of Sciences and Technology, University of the Azores, 9901-862 Horta, Portugal; 3 3B’s Research Group, I3Bs—Research Institute on Biomaterials, Biodegradables and Biomimetics of the University of Minho, Headquarters of the European Institute of Excellence on Tissue Engineering and Regenerative Medicine, AvePark—Parque de Ciência e Tecnologia, Rua Ave 1, Edifício 1(sede), Barco, 4805-694 Guimarães, Portugal; rgreis@i3bs.uminho.pt (R.L.R.); tiago.silva@i3bs.uminho.pt (T.H.S.); 4 ICVS/3B’s—PT Government Associate Laboratory, Braga, 4710-057 Guimarães, Portugal

**Keywords:** *Bathymodiolus azoricus*, hydrothermal vent, innate immunity, mussel adhesive proteins, bioadhesives, byssal collagen, biomineralization, transcriptomic reanalysis, marine biomaterials

## Abstract

The deep sea harbors a substantial proportion of the ocean’s unexplored biological and chemical diversity, while hydrothermal vent ecosystems expose resident organisms to unusual combinations of hydrostatic pressure, steep chemical gradients, reduced compounds, and elevated metal concentrations. This Review examines the deep-sea vent mussel *Bathymodiolus azoricus*, a dominant species at Mid-Atlantic Ridge hydrothermal fields, as a source of biological mechanisms and molecular systems with potential biomedical relevance. We integrate three areas that have largely developed separately in the literature: innate immunity and host–symbiont interactions, mussel-derived wet adhesion and byssal structural proteins, and shell biomineralization and repair. These published observations are complemented by targeted reanalyses of legacy and more recent *B. azoricus* transcriptomic resources, used here as supporting transcriptomic evidence for molecular families relevant to these themes rather than as standalone genome-scale transcriptomic studies. Particular attention is given to mussel foot proteins and byssal collagens as candidate templates for wet-tissue adhesives and structural biomaterials, and to shell-derived calcium carbonate as a potential precursor for calcium-phosphate-based materials. We further advance a specific, testable hypothesis—long-term exposure to the metal-rich hydrothermal vent environment may have influenced the metal-binding chemistry of *B. azoricus* adhesive and structural proteins, potentially generating functional properties distinct from those of shallow-water mytilids. This possibility is biologically plausible in light of established DOPA–metal coordination mechanisms in mussel adhesion, but no direct comparative measurements of Fe^3+^-binding affinity, metal-mediated cross-linking, or adhesive performance currently demonstrate such an advantage in *B. azoricus*. The species should therefore be regarded not as a proven source of superior vent-adapted biomaterials, but as a well-suited experimental system in which immunity, bioadhesion, biomineralization, and environmental adaptation converge to generate specific hypotheses for biomedical discovery. Comparative functional studies, protein-level validation of transcript-derived candidates, and improved molecular characterization of foot and mantle tissues will be required to test these possibilities.

## 1. Introduction

The oceans cover more than 70% of the Earth’s surface and harbor an immense diversity of life. Over millions of years of evolution, marine organisms have developed adaptations that enable survival under a wide range of extreme physical and chemical conditions. Deep-sea hydrothermal vents represent one of the most chemically and physically challenging marine environments. They occur worldwide in association with volcanic and tectonic activity and support chemosynthesis-based ecosystems shaped by high hydrostatic pressure, steep thermal gradients, and elevated concentrations of sulfur compounds, methane, and metals [1,2]. These systems sustain diverse communities of endemic invertebrates adapted to such physicochemical extremes.

Among the macrofauna inhabiting deep-sea hydrothermal vents, the mussel *Bathymodiolus azoricus* is a dominant species along much of the Mid-Atlantic Ridge (MAR), where it typically forms dense aggregations. Its successful colonization of sulfide- and methane-rich habitats is enabled primarily by a dual endosymbiosis with sulfur-oxidizing and methane-oxidizing bacteria housed within specialized gill epithelial cells [3,4]. While this symbiosis is considered the principal mechanism supporting host nutrition in these environments [5], *B. azoricus* must also maintain cellular, structural, immune, and repair functions under high hydrostatic pressure, permanent darkness, fluctuating chemical conditions, and exposure to hydrothermal-fluid-derived metals [6,7]. Such conditions provide strong evolutionary and physiological contexts in which molecular systems involved in stress tolerance, immunity, metal homeostasis, tissue repair, and structural integrity may be shaped. Whether individual *B. azoricus* molecules consequently possess functional properties distinct from homologues in shallow-water mussels, however, must be established experimentally rather than inferred from habitat alone.

The potentially distinctive biological and biochemical features of molecules derived from organisms inhabiting extreme marine environments have attracted increasing interest as a potential source of pharmaceuticals, cosmeceuticals, and biomaterials, particularly for regenerative medicine, tissue engineering, and wound-healing applications [8,9]. Hydrothermal vent animals and their associated microbial communities therefore represent a comparatively underexplored source of marine-derived molecules and materials with potential relevance to human health [10,11,12]. The biomedical potential of *B. azoricus* is not restricted to structural biomaterials: bathymodiolamides C, D, and E have been isolated directly from this species and reported to induce necrotic cell death [13], providing a species-specific example of bioactive natural-product chemistry within the wider field of mollusc-derived natural products [14].

In 2010, Bettencourt and colleagues published the first high-throughput transcriptome of a deep-sea hydrothermal vent animal, based on the gill tissue of *B. azoricus* [10]. The resulting DeepSeaVent database was originally developed to address a specific biological question: how does the immune system of an animal simultaneously defend against pathogens while maintaining permanent and beneficial bacterial symbioses? This immunological motivation has received comparatively little attention as the dataset subsequently became a resource for broader biomolecular discovery.

In this Review, we reconnect the immunological and biomaterials perspectives that have largely developed separately around *B. azoricus*. We first examine its established role as a model for innate immunity and host–symbiont interactions, then consider the molecular and structural systems with potential relevance to biomedical materials, including mussel foot adhesive proteins, byssal collagen, and shell biomineralization and repair. Across these themes, we distinguish evidence demonstrated directly in *B. azoricus* from transcriptomic observations obtained through targeted reanalysis, functional knowledge derived from shallow-water mussels, and hypotheses that remain to be experimentally tested. Within this framework, we advance a specific hypothesis: that long-term exposure to the metal-rich hydrothermal vent environment may have influenced the metal-binding chemistry and structural properties of *B. azoricus* adhesive and byssal systems. In particular, Fe^3+^-dependent catechol chemistry provides a mechanistically plausible basis for asking whether vent-mussel adhesive proteins differ functionally from their shallow-water homologues. This possibility is not presented as a demonstrated advantage; direct comparative measurements of metal binding, cross-linking, and adhesive performance are required to test it.

To support this critical synthesis, we incorporate targeted reanalyses of three *B. azoricus* transcriptomic resources: the original 2010 454 gill dataset, an independently generated 2024 Illumina gill transcriptome, and a foot-tissue transcriptome. These analyses were undertaken to interrogate specific molecular families directly relevant to the biological and biomedical questions addressed in this Review, rather than as standalone genome-scale transcriptomic studies. Accordingly, transcriptomic observations are used as supporting molecular evidence for candidate identification and hypothesis generation and are not interpreted as demonstrating protein-level expression, functional activity, or superior biomaterial performance. The analytical procedures and limitations of each dataset are described in Box 1 and Box 2.

Box 1 **Targeted reanalysis of the 2010 and 2024 gill transcriptomic resources.**

  Raw 454 reads from the archived 2010 dataset (SRR067874; 561,233 reads retrieved) were quality-trimmed with cutadapt by removing adapter and poly-A/poly-T sequence, applying a Q20 quality cutoff and a minimum retained length of 100 bp; 532,555 reads remained after trimming. The retained reads were assembled de novo with MIRA v5.0.0rc2, yielding 26,363 contigs (9.9 Mb total; mean length 376 bp). Coding regions were predicted with TransDecoder using LongOrfs followed by Predict with --single_best_only, yielding 4949 predicted proteins from 5812 candidate ORFs. Predicted proteins were searched against SwissProt using DIAMOND blastp (E-value ≤ 1 × 10^−5^), returning 2786 hits (56.3% of predicted proteins), and were additionally annotated using eggNOG-mapper v2.1.13 (3766 proteins with annotations) and InterProScan v5.78-109.0 (26,324 domain matches). Annotation outputs were consolidated into a relational SQLite database linked through protein and contig identifiers to permit targeted keyword-, homology-, and domain-based interrogation.

  The 2024 gill transcriptome was processed from an already-assembled Trinity dataset comprising 212,318 transcripts. Coding regions were predicted with TransDecoder, yielding 44,389 predicted proteins, and annotation was performed using Trinotate v4, including DIAMOND blastp/blastx searches against SwissProt (E-value ≤ 1 × 10^−5^) and hmmscan searches against Pfam-A. Results were loaded into an independently queryable SQLite database. The two gill resources were interrogated for the same biological target set summarized in Table 1A,B, using a common significance threshold where homology searches were applied and deduplication by base transcript identifier where appropriate. Because the underlying sequencing platforms, assemblies, annotation workflows, and some family-specific searches differ, the resulting candidate counts should not be interpreted as direct quantitative measures of differential expression, gene copy number, or family expansion between the datasets.

Box 2 **Foot-tissue reanalysis and targeted homology-search methodology.**

  Unlike the gill datasets, foot-tissue sample N1188 was available as an already-assembled transcriptome (Trinity, longest-isoform representative per gene; 927,084 sequences) rather than raw reads requiring de novo assembly. Coding regions were predicted with TransDecoder using the default minimum ORF length of 100 aa, yielding 48,778 predicted proteins, and were annotated using the Trinotate v4 toolchain applied to the 2024 gill dataset (DIAMOND blastp/blastx against SwissProt, E ≤ 1 × 10^−5^; hmmscan against Pfam-A; results loaded into a queryable SQLite database).

  The standard SwissProt/TransDecoder workflow initially returned no named Mfp or preCol candidates. Two methodological factors were identified that could account for this failure: most characterized Mfp and preCol reference sequences are represented in UniProt/TrEMBL rather than the reviewed SwissProt subset, and the default TransDecoder minimum ORF length can exclude short candidate ORFs. A targeted ref-erence panel was therefore assembled (Mfp-1, Q25460/Q27409; Mfp-2, Q25464; Mfp-3, Q9GUX7/Q9NAT8; Mfp-5, E7DRN3; preCol-D, O44367; preCol-NG, A9QLN3; preCol-P, O16161) and searched directly against the transcript sequences using DIAMOND blastx with a relaxed initial threshold (E ≤ 1 × 10^−3^; sequence masking disabled) to retain sensi-tivity to short, repetitive, and cross-genus-divergent candidates. Candidates reported in the manuscript were subsequently restricted to alignment length ≥ 50 aa and E ≤ 1 × 10^−5^. Candidates lacking a TransDecoder-predicted ORF were translated from the nucleotide alignment coordinates for inspection.

**Table 1:** (**A**) Immune-, extracellular-matrix-, and wound-repair-associated molecular targets recovered from the 2010 and 2024 *B. azoricus* gill transcriptomic resources. Negative or rejected candidates are retained where they clarify search specificity; candidate counts are not interpreted as direct quantitative comparisons between datasets. (**B**) Growth-factor-, cytokine-, and signaling-associated molecular targets recovered from the 2010 and 2024 *B. azoricus* gill transcriptomic resources. The two EGF searches addressed different levels of domain specificity and should not be interpreted as directly comparable counts.

**(A)**			
**Gene/Family**	**2010 Reanalysis (SRR067874)**	**2024 Illumina Reanalysis**	**Interpretation/Notes**
Integrin	6 candidate proteins, including c6448 (integrin alpha), c2540, c22446, c11545, c3135, c4697	32 transcripts recovered	Recovered in both datasets; the larger 2024 count is not interpreted as a paralog or expression comparison.
Tissue inhibitor of metalloproteinase (TIMP)	7 candidate proteins: TIMP1 (c1670), TIMP3 (c607), TIMP4 (c2320, c529), c1316, c2667, c2355	19 proteins with Pfam TIMP-domain support (E ≤ 1 × 10^−5^)	Recovered in both datasets.
Matrix metalloproteinase (MMP)	c1756, annotated as MMP3/stromelysin	11 transcripts recovered	A bacterial MMPL-transporter substring match (c1327) was excluded from the 2010 screen.
Cadherin	7 candidate proteins: c1449, c21305, c23457, c3332, c1160, c5501, c18246	74 transcripts recovered	Recovered in both datasets; counts are not treated as quantitative family-size estimates.
Collagen/proteoglycan	Collagen: 68 hits across multiple collagen classes, not narrowed to type IV. Proteoglycan: three weak related hits; no proteoglycan core protein recovered.	Collagen type IV: three transcripts with alpha-1(IV)/alpha-2(IV)-like assignments. Proteoglycan: three transcripts including a basement-membrane heparan-sulfate-proteoglycan-like core protein.	The 2024 data provide stronger candidate support for type-IV collagen and a core proteoglycan, but do not independently confirm aggrecan specifically.
Cathepsin	13 candidate proteins including cathepsin B, C, L1, S, and Z-like assignments	23 proteins with Pfam support (E ≤ 1 × 10^−5^)	Recovered in both datasets.
Coagulation factor/plasminogen/fibrinogen	Coagulation-related: c3251 (factor 5/8 type C domain). Plasminogen-related: three Kringle-4-domain proteins. Fibrinogen-related: 35 proteins with fibrinogen C-terminal domains.	15 coagulation-related transcripts; 14 plasminogen-related transcripts; 25 fibrinogen-related transcripts	A Translin-associated-factor-X substring match was excluded from the 2010 coagulation screen; the remaining families were recovered in both datasets.
Sialic acid-binding lectin (Siglec)	5 proteins, including SIGLEC1-like candidates c3185 and c1725	No Siglec/sialoadhesin candidate recovered under the applied criteria	The 2010 candidate-family recovery was not reproduced in the 2024 dataset and is retained as an open discrepancy.
Allograft inflammatory factor 1 (AIF1)	c3500, annotated as allograft inflammatory factor 1 with paired EF-hand domains	71 proteins recovered by the applied Pfam search	AIF1-associated candidates were recovered in both datasets; counts are not interpreted as family expansion.
**(B)**			
**Gene/Family**	**2010 Reanalysis (SRR067874)**	**2024 Illumina Reanalysis**	**Interpretation/Notes**
EGF (epidermal growth factor)	29 proteins bearing EGF-like or vWF-D+EGF domains in a broad structural-domain sweep	3 proteins recovered using ligand-focused EGF-domain criteria (HBEGF_C/EGF_TGFA-like/EGF_Epiregulin-like)	Methodologically distinct searches; both support EGF-domain-containing candidates but the counts are not directly comparable.
Transforming growth factor beta (TGF-β)	7 proteins, including c7174 with TGF-β propeptide/ligand-domain support	9 proteins with Pfam TGF_beta/TGFb_propeptide support (E ≤ 1 × 10^−5^)	Candidates recovered in both datasets.
Fibroblast growth factor (FGF)	c7365 corresponds to FGFR4 rather than an FGF ligand	3 proteins with Pfam FGF-ligand-domain support (E ≤ 1 × 10^−5^)	The 2010 result is receptor-associated rather than ligand evidence; ligand-domain candidates were recovered in 2024.
Tumor necrosis factor (TNF) superfamily	78 proteins, predominantly C1q-TNF-related proteins and LITAF-associated assignments	28 proteins recovered using Pfam TNF/TNFSF4_N criteria (E ≤ 1 × 10^−5^)	Neither dataset is interpreted as evidence for classical mammalian TNF-α; the searches recover bivalve TNF/C1q-related candidates under different criteria.
VEGF/IGF/HGF	VEGF: three weak pathway-associated annotations without a VEGF/VEGFR protein; IGF: two weak Somatomedin-B-domain hits in larger proteins; HGF: no candidate recovered	VEGF: none; IGF: none; HGF: none under the applied criteria; one bacterial HGF-like DIAMOND v2.2.5 (blastp/blastx mode) hit was excluded	No convincing ligand candidate for these three families was recovered in either dataset under the applied criteria.
EPAS1/HIF-2α	One indirect HIF-pathway-associated hit (c7199; HIF1AN/FIH-1-related), not EPAS1 itself	One EPAS1-like candidate: TRINITY_DN117996_c0_g1_i1, 32.7% identity, E = 1.89 × 10^−8^	The 2024 dataset contains a homology-supported EPAS1-like candidate; the 2010 hit is retained only as an indirect pathway-associated observation.

## 2. Innate Immunity and Host–Symbiont Responses in *Bathymodiolus azoricus*

Before considering the structural and adhesive biomaterials associated with *B. azoricus*, it is important to recognize that the species already has an established biomedical relevance as an experimental system for innate immunity and host–microbe interactions. Its permanent association with dense populations of chemosynthetic bacterial endosymbionts requires the host to accommodate beneficial microorganisms while retaining the capacity to recognize and respond to potentially harmful microbes. This biological paradox has motivated a substantial body of experimental work on hemocyte function, immune signaling, antimicrobial responses, and host–symbiont interactions in *B. azoricus* [7,15,16,17,18]. The resulting evidence provides a direct biological foundation for the immunological component of this Review, distinct from the more exploratory hypotheses concerning the functional properties of vent-derived biomaterials.

Marine bivalves rely on cellular and humoral components of innate immunity to recognize and respond to microorganisms. In *B. azoricus*, the hemolymph and extrapallial fluid contain three hemocyte types, with granulocytes representing the most abundant and principal phagocytic population; their surface carbohydrate epitopes were further characterized using fluorescent WGA-lectin staining [15]. Exposure of hemocytes to microbial elicitors, including zymosan, glucan, *Bacillus subtilis*, and *Vibrio parahaemolyticus*, was associated with activation of the MAPK pathways ERK, p38, and JNK, as assessed by Western blotting. Ca^2+^-dependent cellular activation was also demonstrated using the fluorescent indicator Fura-2 AM following exposure to laminarin or live *V. parahaemolyticus* [15]. In vivo exposure to *Vibrio* additionally induced expression of the antimicrobial peptide gene *mytilin* in hemocytes associated with the gill epithelium, providing direct evidence that a defined immune effector responds to bacterial challenge in this species [15].

This line of research was extended through bacterial-challenge experiments using *Vibrio diabolicus*, a species originally isolated from the hydrothermal-vent polychaete *Alvinella pompejana* and therefore a vent-associated bacterium of particular ecological relevance for experimental challenge studies. Across acclimatization and challenge experiments, genes involved in pattern recognition, signal transduction, stress responses, and immune effector functions showed time-dependent expression patterns that were examined in relation to changes in endosymbiont colonization. Comparisons between mussels originating from the Menez Gwen and Lucky Strike hydrothermal fields further revealed site- and tissue-associated differences in the expression of genes including *Carcinolectin*, *Serpin-2*, SRCR, IRGs, RTK, TLR2, NF-κB, HSP70, and ferritin [16,17,18].

Endosymbiont-associated genes, including ALDH, CA, CBB, MeDH, MMO, and SOXB, also showed altered expression following *Vibrio* challenge, demonstrating that bacterial challenge is accompanied by transcriptional responses in both host and symbiont compartments. These observations support a coordinated host–symbiont response rather than two entirely independent processes [7,16,17,18]. Whether increased symbiont activity contributes directly to host defense, metabolic compensation, or more general physiological resilience remains unresolved and should therefore be regarded as a mechanistic hypothesis rather than an established immune function of the symbionts.

Taken together, these studies establish *B. azoricus* as a useful experimental system for investigating innate immunity, microbial recognition, stress responses, and the regulation of host–symbiont coexistence in a deep-sea animal [7,15,16,17,18]. From a biomedical-discovery perspective, characterized or candidate molecular classes in this system include antimicrobial peptides such as mytilin, pattern-recognition and signaling components, cytokine-like molecules, and regulators of cellular stress and apoptosis. These molecules may provide candidates for future antimicrobial or recognition-based applications and, more broadly, comparative tools for investigating mechanisms that permit persistent host–microbe coexistence. Their biomedical utility, however, cannot be inferred from sequence identity or ecological context alone and requires molecule-specific functional characterization.

Direct comparison of immune responses between *B. azoricus* and the shallow-water mussel *Mytilus galloprovincialis* has already provided an experimental framework for examining species-level differences under bacterial challenge [18]. Nevertheless, whether individual antimicrobial peptides, pattern-recognition molecules, or other immune effectors from *B. azoricus* possess biochemical or functional properties that distinguish them from shallow-water homologues remains unresolved. Long-term coexistence with dense and metabolically active bacterial endosymbionts provides a biologically compelling context in which to investigate such differences, but does not itself demonstrate them. Comparative molecular and functional studies will therefore be required before vent-specific immune properties can be linked to biomedical applications.

## 3. Transcriptomic Resources and Targeted Reanalysis in *Bathymodiolus azoricus*

The 2010 gill transcriptome of *B. azoricus* represented the first high-throughput tissue transcriptome reported for a deep-sea hydrothermal vent animal [10]. It generated a searchable sequence resource that supported gene-expression studies in this non-model species and substantially expanded the available catalog of candidate genes associated with immunity, cell adhesion, extracellular-matrix biology, tissue repair, biomineralization, and host–microbe interactions [7,10]. Although developed principally to investigate innate immunity and host–symbiont biology, the dataset subsequently became useful for identifying molecular families relevant to broader biological and biomedical questions.

The DeepSeaVent web portal originally developed to query the 2010 dataset [10] is no longer accessible because the server hosting its custom browser and BLAST interface has been discontinued. The underlying sequencing reads remain available through the NCBI Sequence Read Archive (SRA accession SRR067874). For the present Review, these archived reads were independently reprocessed and reannotated to recover transcriptomic information relevant to the molecular families considered here. The purpose of this reanalysis was not to reproduce the original DeepSeaVent assembly exactly, but to generate a contemporary, queryable representation of the archived dataset using the workflow summarized below and detailed in Box 1.

A second, independently generated gill transcriptome was produced in 2024 using Illumina sequencing. This dataset provides an independent sequence resource from the same species and tissue and is used here to examine whether molecular families identified in the reprocessed 2010 dataset are also represented in a more recent transcriptome generated using a different sequencing platform. It therefore provides cross-dataset corroboration of candidate-family recovery rather than functional or protein-level validation. The dataset is publicly available through the NCBI Sequence Read Archive under BioProject PRJNA1508204 (SRA accession SRR40024054; BioSample SAMN62218460).

The transcriptomic analyses incorporated here followed complementary workflows according to the form in which each dataset was available. The archived 2010 gill dataset was reprocessed from raw 454 reads through quality trimming, de novo assembly, coding-region prediction, and functional annotation. The independently generated 2024 gill transcriptome was available as a preassembled Trinity transcript set and was therefore subjected to coding-region prediction and annotation without repeating de novo assembly. Both resources were subsequently interrogated for the molecular targets summarized in Table 1A,B. Because the two datasets differ in sequencing technology, assembly depth, annotation workflow, and, for some target families, search strategy, comparisons are interpreted primarily as evidence for the presence or non-recovery of candidate molecular families rather than as quantitative comparisons of transcript abundance or gene-family size. The foot-tissue transcriptome, discussed later, was also available as a preassembled Trinity dataset and was subjected to a related annotation workflow followed by a targeted reference-guided search for mussel foot proteins and preCol sequences (Box 2).

A more focused comparison can be made for the three targets that were experimentally examined by RT-PCR/qPCR in the original 2010 study: integrin, EGF, and aggrecan (Table 2). The present reanalyses recover transcriptomic evidence belonging to the corresponding integrin and EGF-associated molecular families, although this does not constitute experimental revalidation of the original transcripts. Aggrecan itself was not recovered from the reprocessed 2010 assembly. The 2024 dataset contains core proteoglycan candidates, including a basement-membrane heparan-sulfate-proteoglycan-like sequence, but this should not be interpreted as independent confirmation of aggrecan specifically without a direct sequence assignment. The failure to recover the original aggrecan transcript in the 2010 reassembly may reflect differences in assembly depth or analytical workflow, but the cause cannot be established from the available data.

Taken together, the two gill resources provide complementary transcriptomic support for several molecular families relevant to immunity, extracellular-matrix remodeling, and tissue repair, while also revealing non-recovered and method-dependent targets that should not be obscured. Their principal value in this Review is therefore not to provide quantitative cross-transcriptome comparisons, but to identify and prioritize candidate molecular families for subsequent functional investigation. Experimental evidence from the original studies and transcriptomic evidence from the present reanalyses are treated separately throughout the sections that follow.

## 4. Shell Biomineralization, Repair, and Biomedical Potential in *Bathymodiolus azoricus*

Molluscan shells are biomineralized composites composed predominantly of calcium carbonate (CaCO_3_) deposited within a small but functionally important organic matrix of proteins, polysaccharides, and other macromolecules [19]. In nacre-forming species, this organic–inorganic organization produces a hierarchical material with distinctive mechanical properties that has attracted considerable interest in biomaterials research. Nacre and nacre-derived materials have shown favorable interactions with bone-forming cells and have consequently been investigated as natural templates or raw materials for bone-graft applications [19,20].

Bivalve-shell CaCO_3_ can also serve as a precursor for calcium-phosphate biomaterials. Macha et al. converted shells of the shallow-water mussel *Mytilus galloprovincialis* into monetite (dicalcium phosphate) through a hydrothermal conversion process [21], demonstrating the feasibility of transforming mussel-shell CaCO_3_ into a calcium-phosphate phase relevant to bone-repair materials. Comparable conversion has not, to our knowledge, been tested using *B. azoricus* shell. Whether its shell chemistry influences conversion efficiency, resulting phase composition, crystallinity, trace-element content, or resorption behavior therefore remains an experimental question.

Shell formation and repair are controlled primarily by the mantle and by cellular responses recruited following injury. The mantle comprises epithelial and connective tissues involved in secretion of shell-matrix components and regulation of biomineral deposition, and mantle transcriptomes from several bivalves, including *Pecten maximus*, *Mytilus edulis*, and *Crassostrea gigas*, have revealed genes associated with biomineralization and extracellular-matrix organization [22,23,24]. Following tissue or shell injury, hemocytes can accumulate at damaged sites and participate in wound sealing, extracellular-matrix remodeling, and subsequent regeneration. Rapid repair and regenerative responses have been documented in other bivalves, including *Scrobicularia plana* and *Donax serra* [25,26], providing comparative evidence that wound repair is an active and coordinated process across multiple bivalve lineages.

In *B. azoricus*, shell damage provides a particularly useful point of convergence between the immunological and biomaterials themes of this Review. Experimental shell injury has demonstrated recruitment of hemocytes to repair sites and transfer of CaCO_3_ during shell regeneration, while mantle-to-shell calcium transfer is measurably affected by hydrostatic pressure [27,28]. These observations provide direct evidence that immune-associated cellular responses, biomineralization, and environmental conditions interact during shell repair in this species.

Tyrosinase/phenoloxidase pathways provide a further possible mechanistic link between immunity, wound repair, and shell formation. Phenoloxidases catalyze oxidation of phenolic substrates to quinones that can subsequently participate in melanization and protein cross-linking, and prophenoloxidase-associated pathways contribute to immune and wound responses in several invertebrate groups [29,30]. Tyrosinase gene-family expansion and mantle-associated expression have also been documented in bivalves, supporting roles for these enzymes in shell formation and pigmentation [31].

**Figure 1:**
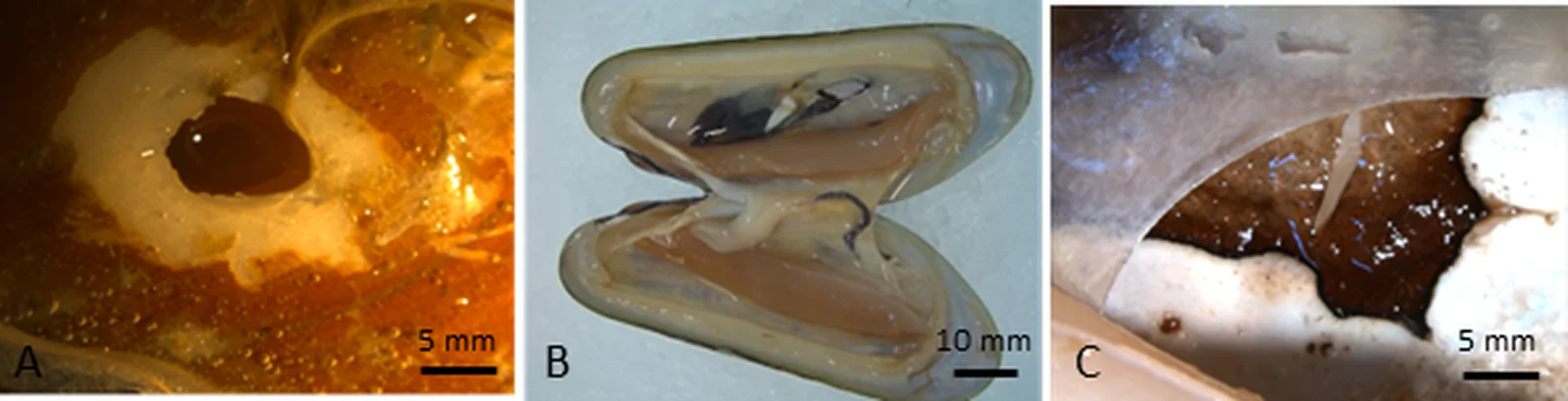
Preliminary observation of the shell response following mechanical injury in *Bathymodiolus azoricus*. (**A**) Mechanical opening produced in the shell without intentional disruption of the underlying mantle tissue. (**B**) Dark deposition developing around the injured region. (**C**) Higher-magnification view of the deposition surrounding the shell lesion. The pigmentation is consistent with a melanization-associated wound response and is compatible with involvement of phenoloxidase-related chemistry; however, the chemical identity of the deposited material and phenoloxidase activity were not determined directly in these preliminary observations. Scale bars, 5 mm (**A**,**C**) and 10 mm (**B**).

Preliminary shell-injury observations in *B. azoricus* revealed conspicuous dark deposition around mechanically damaged shell regions (Figure 1). The appearance is consistent with a melanization-associated wound response and is therefore compatible with involvement of phenoloxidase-related chemistry; however, these observations were qualitative and did not include biochemical identification of the deposited material or direct measurement of phenoloxidase activity. They should consequently be regarded as illustrative preliminary observations rather than mechanistic evidence. Dedicated enzymatic, histochemical, and molecular analyses will be required to determine whether tyrosinase/phenoloxidase pathways participate directly in *B. azoricus* shell repair.

Trace-element analyses provide direct evidence that the geochemical environment is reflected in *B. azoricus* shell composition. Shells from hydrothermal-vent specimens contain micro-essential metals associated with the surrounding vent environment [32]. This demonstrates that environmental geochemistry can leave a measurable compositional signature in a structural tissue of *B. azoricus*. It does not, however, establish that analogous metal exposure has altered the binding properties of adhesive proteins or other biomolecules.

Direct structural characterization further demonstrates that environmental pressure affects the integrity of *B. azoricus* nacre. SEM analysis across three Mid-Atlantic Ridge vent fields found nacre micromorphology to be broadly conserved despite differences among sites, with observed variation associated more closely with Ca^2+^ availability than with pressure [33]. In experimental maintenance conditions, decompression was associated with rapid nacre dissolution: shell deterioration became detectable after exposure to atmospheric pressure and progressed over time, whereas specimens maintained under elevated pressure were comparatively protected [33]. These observations provide direct evidence that the structural stability of *B. azoricus* shell is pressure-sensitive. Whether this pressure dependence reflects specific evolved molecular adaptations of the shell matrix requires further comparative investigation.

From a biomaterials perspective, these findings make *B. azoricus* shell scientifically interesting, but they do not yet demonstrate superior biomedical performance. Vent-associated trace-element incorporation and pressure-sensitive nacre stability raise testable questions about whether *B. azoricus* shell differs from shallow-water mussel shell in conversion behavior, mechanical properties, cytocompatibility, degradation, or ion release after processing into calcium-phosphate materials. These properties must be evaluated directly before any biomedical advantage can be inferred.

A major molecular knowledge gap remains. Unlike gill and foot tissues, the *B. azoricus* mantle has not yet been characterized through a dedicated transcriptomic analysis. Consequently, the shell-matrix proteins, calcium-transport systems, tyrosinases, and regulatory pathways associated with biomineralization and repair remain incompletely characterized in this species. A mantle-focused transcriptome, ideally combined with proteomic analysis of shell matrix and experimentally defined repair stages, would provide a necessary basis for linking sequence information to shell formation, environmental responses, and biomaterial properties.

## 5. Mussel Foot Adhesion and Bioadhesive Potential in *Bathymodiolus azoricus*

Marine-derived chitin, chitosan, alginate, hyaluronic acid, and broadly sourced collagens have been extensively reviewed as wound-healing materials and are not specific to mussels [9]. The discussion here therefore focuses on features directly relevant to *B. azoricus* and mussel-inspired biomaterials: byssal adhesive proteins, catechol-based wet adhesion, and the structural components of the byssus. Their biomedical relevance is considered beyond cutaneous wound repair, particularly in the context of wet-tissue adhesives and surgical sealants, where reliable adhesion under hydrated conditions remains a major materials challenge [34,35].

Marine mussels attach to submerged surfaces through the byssus, a fibrous structure comprising collagenous threads that terminate in protein-rich adhesive plaques. The byssus is produced by specialized glands within the foot, where adhesive and structural proteins are secreted, organized, and cured before attachment to the substrate [36,37,38,39,40,41]. This system has become one of the principal biological models for understanding strong adhesion under wet conditions.

**Figure 2:**
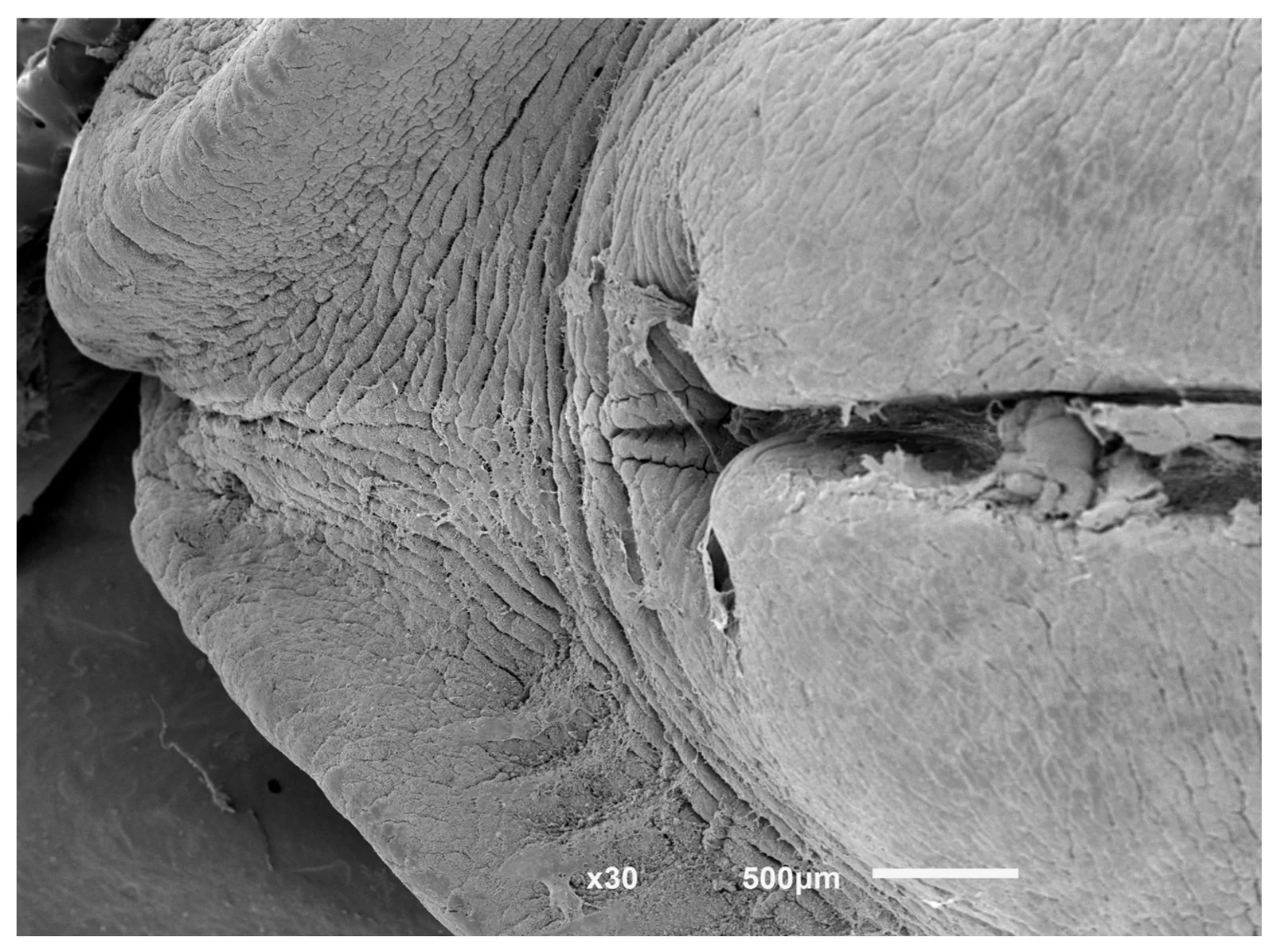
Scanning electron micrograph of the foot of *Bathymodiolus azoricus*. The prominent central fissure corresponds to the byssal groove/distal depression associated with formation and positioning of the byssal apparatus. Transverse rugae across the muscular foot permit expansion and movement during substrate engagement and byssus deposition. Proteinaceous or mucous material is visible locally within the groove, although its precise stage of byssus formation was not determined from the image alone. Image courtesy of Dr. Isabel Leonor, 3B’s Research Group. Magnification ×30; scale bar, 500 μm.

**Figure 3:**
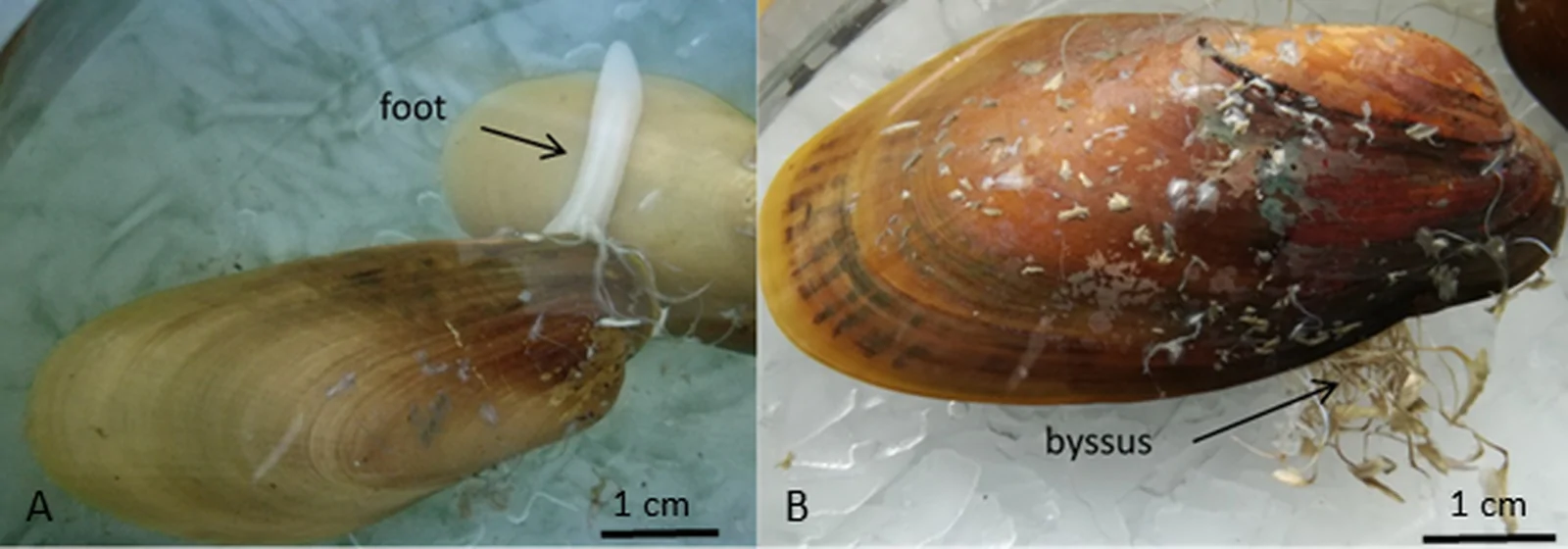
Foot and byssus of *Bathymodiolus azoricus*. (**A**) The extended foot (arrow), a muscular, tongue-like organ used for locomotion and byssus deposition, protruding from between the valves. (**B**) Ventral view showing byssal threads (arrow) secreted by the foot’s byssal gland, anchoring the animal to the substrate. The shell displays the characteristic brown coloration of *B. azoricus*, an oblong outline, and concentric growth lines; irregular marks on the shell surface are consistent with sites of former byssal-plaque adhesion from neighboring mussels. Scale bars, 1 cm.

*Bathymodiolus azoricus* retains the characteristic mytilid byssal attachment system and remains firmly anchored to hard substrata at hydrothermal vent sites. Its muscular foot contains the byssal groove and associated secretory regions involved in byssus formation (Figure 2 and Figure 3). Unlike shallow-water mytilids, however, *B. azoricus* operates this attachment system under high hydrostatic pressure and within a chemically dynamic hydrothermal environment. These ecological conditions provide the rationale for investigating its adhesive proteins comparatively, but they do not by themselves demonstrate altered adhesive performance.

A defining feature of extensively characterized mussel adhesive proteins from shallow-water mytilids is the post-translational conversion of selected tyrosine residues to 3,4-dihydroxyphenyl-L-alanine (DOPA). Catechol groups contributed by DOPA mediate both interfacial interactions with surfaces and cohesive cross-linking within the adhesive matrix, including coordination with metal ions [42,43]. This chemistry has inspired a wide range of synthetic and recombinant wet-adhesive systems. Importantly, although the present transcriptomic analysis identifies an Mfp-2-like candidate in the *B. azoricus* foot, the extent and distribution of tyrosine hydroxylation and DOPA incorporation in native *B. azoricus* foot proteins have not yet been established experimentally. The presence of an Mfp homolog should therefore not be interpreted as direct biochemical demonstration of DOPA content or metal-binding behavior in this species.

Mussel-inspired adhesion is particularly relevant to biomedical settings in which conventional closure methods or synthetic adhesives perform poorly in the presence of water, blood, or other biological fluids. Catechol-functionalized polymers and recombinant MAP-based systems have therefore been investigated as tissue adhesives, injectable hydrogels, surface coatings, and scaffolds [44,45,46,47]. These approaches seek to combine strong wet adhesion with cytocompatibility and controlled degradation while avoiding limitations associated with existing biological and synthetic adhesives [34,48,49,50]. Recent work has reinforced the translational breadth of mussel-inspired adhesion, including recombinant MAP production, injectable catechol-functionalized hydrogels, tissue adhesives, and engineered protein systems for regenerative and therapeutic applications [51,52,53,54,55,56,57]. These developments provide the broader technological context in which *B. azoricus* Mfp-like candidates should be evaluated, but they do not themselves establish any functional advantage of vent-derived proteins.

Recombinant production is particularly attractive because harvesting native MAPs at useful scale is impractical. However, heterologous expression introduces substantial challenges, including protein solubility and folding, yield, and reproduction of post-translational modifications that contribute to native adhesive function [58]. Synthetic catechol functionalization can circumvent some of these limitations, but such materials reproduce selected chemical principles of mussel adhesion rather than the full molecular architecture of the native proteins.

**Figure 4:**
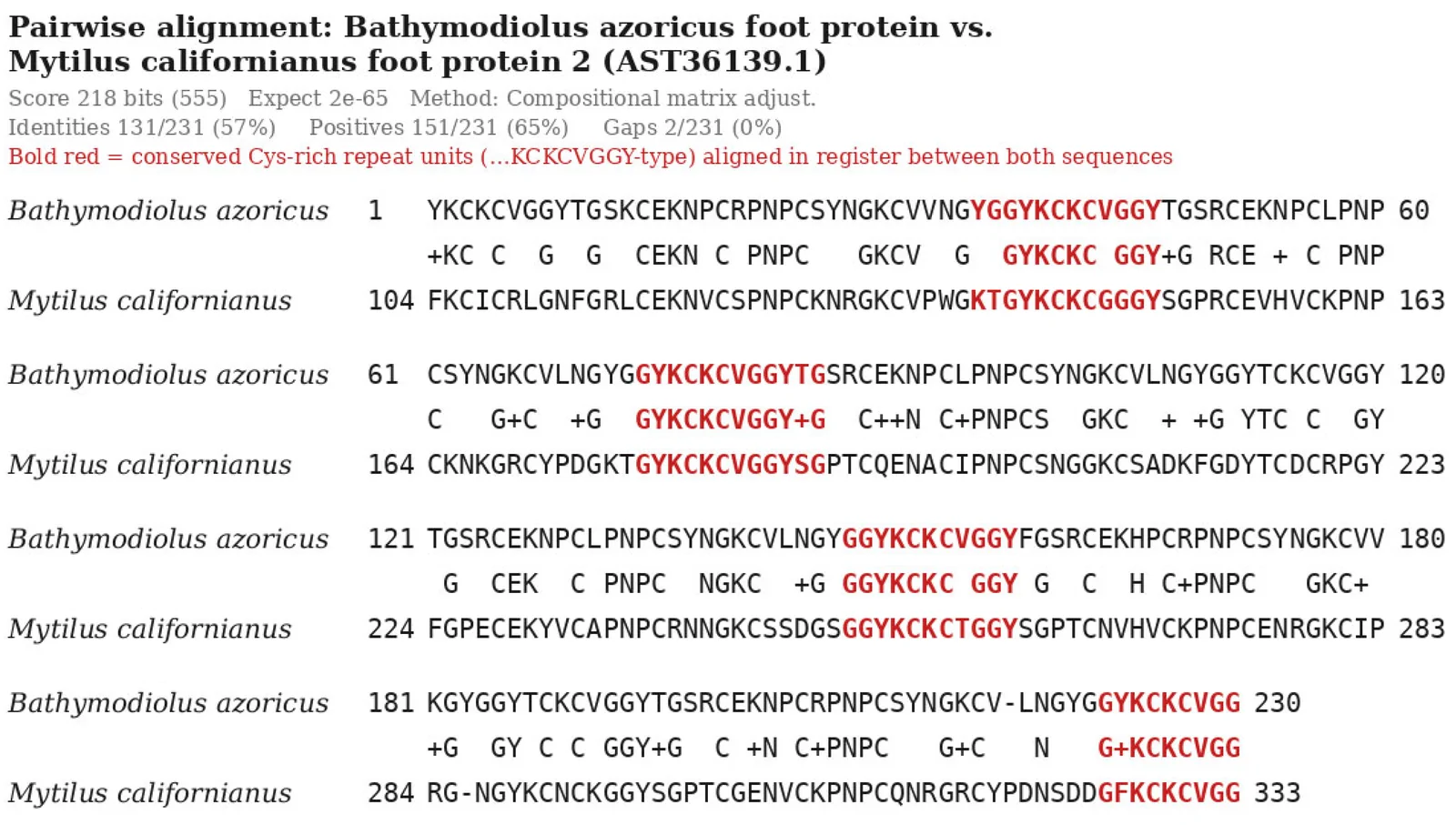
Pairwise sequence alignment supporting an Mfp-2-like candidate in the *Bathymodiolus azoricus* foot transcriptome. The 231-residue translated sequence identified during the original 2017–2019 analysis is aligned with *Mytilus californianus* foot protein 2 (GenBank AST36139.1), showing 57% identity and 65% positive substitutions (E = 2 × 10^−65^). Repeated cysteine-rich motifs associated with the EGF-like repeat architecture of Mfp-2 are highlighted. Targeted reanalysis of the surviving foot transcriptome independently recovered closely related Mfp-2-like candidates, including TRINITY_DN360524. The alignment provides homology-based support for an Mfp-2-like transcript but does not establish protein production, post-translational DOPA modification, or adhesive activity.

A foot-tissue transcriptome of *B. azoricus* (sample N1188; StabVida project RNQ201711000080) was originally generated at the 3B’s Research Group to investigate candidate byssal proteins. Preliminary analyses performed between 2017 and 2019 identified an Mfp-2-like sequence showing 57% identity and 65% positive substitutions across a 231-residue alignment to *Mytilus californianus* foot protein 2 (E = 2 × 10^−65^) (Figure 4). The original computational analysis was subsequently lost with the local server on which it had been performed, although the assembled transcript dataset was retained. Targeted reanalysis of that surviving dataset independently recovered Mfp-2-like candidates, including TRINITY_DN360524, which showed 59.3% identity to UniProt Q25464 (E = 1.0 × 10^−30^), and TRINITY_DN351523, which showed 52.6% identity (E = 1.2 × 10^−44^). These findings provide reproducible transcriptomic support for an Mfp-2-like candidate in *B. azoricus* foot tissue, but do not establish protein abundance, post-translational DOPA modification, or adhesive function.

One candidate, TRINITY_DN380823, initially produced a strong EGF-domain-level match but encoded a 2237-residue Notch-family receptor rather than an Mfp-2-like protein [59]. Its exclusion illustrates an important limitation of searches involving modular repeat-containing proteins: strong similarity within a shared EGF-like domain is insufficient to establish full-protein identity. Candidate interpretation therefore incorporated overall sequence length and protein architecture rather than relying on domain-level alignment scores alone.

Mfp-2 is a major component of the adhesive plaque in characterized *Mytilus* species and consists predominantly of tandem EGF-like repeats stabilized by disulfide bonds [42,60,61]. Calcium- and iron-dependent interactions involving Mfp-2 and other plaque components contribute to plaque organization and mechanical behavior in shallow-water mussels [42]. Recovery of an Mfp-2-like transcript from the *B. azoricus* foot therefore identifies a relevant candidate for comparative study, but equivalent metal-binding, plaque abundance, and adhesive function have not yet been demonstrated for the *B. azoricus* protein.

Mechanistic studies in shallow-water mussels provide an important framework for this comparative question. Byssus fabrication has been shown to involve tightly regulated, metal-ion-dependent curing, while catechol processing is spatially compartmentalized in ways that influence whether DOPA residues participate in cross-linking or other chemical roles [62,63]. These findings establish metal-dependent processing as an integral component of characterized mussel adhesion systems. They provide a mechanistic basis for asking whether chronic exposure to hydrothermal geochemistry has influenced the corresponding chemistry in *B. azoricus*, but they do not constitute evidence that such an evolutionary modification has occurred.

The hydrothermal habitat nevertheless raises a specific and experimentally testable comparative question. Because metal-catechol coordination contributes to mussel adhesive curing and because *B. azoricus* experiences a metal-rich geochemical environment, its adhesive proteins may differ from shallow-water homologues in sequence composition, post-translational modification, metal affinity, or metal-mediated cross-linking behavior. At present, however, no direct measurements demonstrate altered Fe^3+^ affinity, DOPA content, cross-linking efficiency, adhesion strength, cohesion, or self-healing performance in *B. azoricus*. The hypothesis advanced here is therefore not that vent-mussel proteins are demonstrably superior, but that hydrothermal adaptation provides a biologically grounded reason to compare their metal-dependent adhesive chemistry experimentally with that of shallow-water mytilids.

If future biochemical characterization confirms useful adhesive properties, recombinant *B. azoricus* Mfp-like proteins could also be explored as engineering modules rather than used in isolation. Studies with shallow-water recombinant MAPs have shown that fusion to RGD and other extracellular-matrix-derived motifs can promote integrin-mediated cell adhesion and provide additional biological functionality [64,65,66]. Recent recombinant strategies further demonstrate that mussel adhesive sequences can function as modular engineering components: Mfp-derived domains have been combined with curli-, gas-vesicle-, spidroin-, and amyloid-associated modules to generate chimeric materials with tunable assembly and underwater-adhesion properties [67,68]. For *B. azoricus*, these studies provide engineering precedents rather than evidence of equivalent performance.

Recombinant MAPs have also been used as platforms for incorporating antimicrobial functionality; MAP-antimicrobial-peptide fusion constructs have shown activity against Gram-negative bacteria under experimental conditions [69]. This modular strategy is conceptually relevant to the dual perspective developed in this Review because *B. azoricus* possesses both adhesive-protein candidates and experimentally studied immune effectors. However, combining these two molecular classes remains a future engineering possibility: the activity, stability, and safety of individual *B. azoricus* immune molecules would first require direct characterization. Catechol-functionalized biomaterials have also achieved substantial wet-tissue adhesion and mechanical performance in ex vivo models [70], illustrating the performance benchmarks against which any future *B. azoricus*-derived adhesive design would ultimately need to be evaluated.

Translation of *B. azoricus*-derived adhesive proteins would face additional challenges beyond those already encountered with shallow-water MAPs. Natural harvesting from a deep-sea species is neither a realistic nor a desirable manufacturing route, making recombinant or synthetic production essential. Candidate sequences must first be confirmed at the protein level and their post-translational modifications characterized, particularly where adhesive function depends on catechol chemistry. Recombinant yield, folding, reproducible DOPA incorporation, sterilization stability, cytocompatibility, degradation products, immunogenicity, batch consistency, and scalable manufacturing would all require systematic evaluation before any clinical application could be considered. Consequently, the near-term value of *B. azoricus* lies primarily in identifying molecular designs and structure–function relationships that can be reproduced through engineered biomaterials rather than in direct extraction of native adhesive proteins.

## 6. Byssal Collagen and preCol Proteins as Structural Biomaterial Templates

Whereas much of the mussel-inspired biomaterials literature has focused on the non-collagenous adhesive proteins concentrated in the byssal plaque, the thread itself contains a structurally important collagenous protein system. The mussel preCol family comprises modular collagen-like proteins that form a major component of the byssal thread core and contribute to its combination of stiffness, extensibility, and mechanical recovery. Their architecture and assembly therefore offer a complementary biomaterials paradigm to the interfacial adhesion provided by Mfps: rather than primarily mediating attachment to a surface, preCols contribute to the load-bearing and energy-dissipating properties of the attachment apparatus [71,72,73]. Whether the corresponding proteins in *B. azoricus* possess equivalent structural or mechanical properties has not yet been established experimentally.

Experimental work in shallow-water mussels provides the principal precedent for considering byssal collagen as a biomaterial resource. Rodríguez et al. extracted collagen from the byssus of *Mytilus chilensis* using a pepsin-assisted procedure and characterized it as a potential marine collagen source [74]. At the molecular level, Suhre et al. identified a collagen-binding matrix protein involved in organization of the preCol core [71], while Priemel et al. demonstrated that byssal precursors undergo rapid hierarchical self-assembly during thread formation [72]. Together, these studies show that mussel byssus is relevant not only as a source of adhesive chemistry but also as a model for rapid organization of collagenous structural materials.

The capacity of mussel byssal threads to recover mechanically following cyclic loading provides a further design principle of potential relevance to biomaterials exposed to repeated deformation [73]. Such behavior is conceptually attractive for load-bearing or mechanically dynamic tissue-engineering applications, but it should be regarded as biomimetic inspiration rather than evidence that native byssal collagen is already suitable for tendon, ligament, or other clinical repair applications.

For *B. azoricus*, direct biochemical and mechanical characterization of individual byssal preCol proteins is still lacking. The foot-transcriptome analysis discussed in the following section provides transcript-supported candidates related to preCol-D, preCol-NG, and preCol-P, but transcript recovery does not establish their abundance, post-translational state, supramolecular organization, or mechanical contribution to the native byssus. The hydrothermal environment provides a rationale for testing whether these proteins differ from shallow-water homologues in sequence architecture, metal interactions, assembly behavior, or mechanical response, but no such differences have yet been demonstrated. Comparative protein-level and mechanical studies are therefore required before vent-specific structural properties can be inferred. From a translational perspective, the most realistic value of *B. azoricus* preCols would likely lie in identifying structural motifs or assembly principles that can be reproduced recombinantly or synthetically, rather than in harvesting native byssal material from a deep-sea species.

## 7. Transcriptomic Evidence for preCol-D-, preCol-NG-, and preCol-P-like Candidates in the *Bathymodiolus azoricus* Foot

Following identification of Mfp-2-like sequences, the same foot-transcriptome dataset was interrogated using reference sequences for the three characterized mussel preCol classes, preCol-D (UniProt O44367), preCol-NG (A9QLN3), and preCol-P (O16161), using the targeted homology-search strategy described in Box 2. A total of 156 transcript matches passed the predefined alignment-length and E-value filters (Table 3). This broad candidate set should not be interpreted as 156 distinct preCol genes: the Trinity assembly may contain related collagen-like transcripts, partial or redundant sequences, and other collagen-family proteins sharing substantial sequence similarity with the reference proteins. The analysis therefore focused on the strongest individual candidates for each preCol class and on whether their translated sequences were consistent with the expected collagen-like architecture.

Collagen-family proteins are characterized by Gly-X-Y-rich regions, but this motif alone is not specific to mussel preCols. Candidate interpretation therefore considered both homology to characterized preCol references and the overall organization of the translated sequence. Large or Gly-X-Y-rich transcripts were not regarded as preCols solely on the basis of collagen-repeat content. Because formal domain annotation and full-length gene-model validation have not yet been completed for the entire candidate set, the broader group of 156 matches is treated here as a screening pool rather than a defined preCol repertoire.

The best-supported preCol-D-like candidate was TRINITY_DN381679_c3_g3_i2 (634 aa; 42.3% identity; E = 1.35 × 10^−70^), whose translated sequence contains an extended Gly-X-Y-rich region across much of its length. For preCol-NG, the strongest candidate was TRINITY_DN373312_c1_g1_i5 (652 aa; 39.3% identity; E = 3.05 × 10^−70^), likewise displaying a prominent collagen-like repeat region and a short cysteine-rich C-terminal sequence. These features are compatible with preCol-like architecture but are not, by themselves, equivalent to formal structural-domain annotation or protein-level confirmation.

The strongest preCol-P-like candidate was TRINITY_DN376395_c3_g3_i5 (905 aa; 51.3% identity; E = 2.29 × 10^−134^). Its translated sequence contains a central Gly-X-Y-rich collagen-like region flanked by compositionally distinct regions enriched in serine, alanine, glycine, and histidine. This organization is consistent with the general tripartite arrangement described for preCol-P proteins from shallow-water mussels [71,72,73] and therefore provides a strong transcript-supported candidate for a *B. azoricus* preCol-P-like sequence. Because the present interpretation is based on homology and direct inspection of the translated sequence rather than formal domain annotation, the candidate should not yet be regarded as a validated full-length preCol-P protein. Confirmation will require improved gene-model resolution, domain-level annotation, and ultimately protein-level characterization.

These findings should be interpreted at the level at which they were obtained. The foot transcriptome is an assembled RNA-sequence resource, and homology-supported transcripts are not equivalent to validated full-length gene models or demonstrated proteins. The current analysis does not establish protein abundance, tissue localization within specific foot glands, post-translational modification, incorporation into mature byssal threads, or mechanical function. Formal domain annotation of the leading candidates and protein-level approaches such as targeted proteomics would substantially strengthen their assignment. Nevertheless, recovery of independent, high-significance candidates related to each of the three major preCol classes provides a rational transcriptomic basis for prioritizing these proteins for future study in *B. azoricus*.

**Table 3:** Best-supported *B. azoricus* foot-transcriptome candidates identified against reference sequences for the three characterized mussel preCol classes. One leading candidate is shown for each reference class from the broader set of 156 transcript matches passing the alignment-length and E-value filters. Candidate assignment is based on sequence homology and inspection of translated sequence composition; architecture descriptions are provisional and do not represent formal Pfam/InterPro annotation or protein-level validation.

Reference preCol Class	Best Candidate Transcript	Length (aa)	Identity	E-Value	Provisional Architecture Note
preCol-D (O44367)	TRINITY_DN381679_c3_g3_i2	634	42.3%	1.35 × 10^−70^	Extended Gly-X-Y-rich region; short C-terminal non-repetitive region.
preCol-NG (A9QLN3)	TRINITY_DN373312_c1_g1_i5	652	39.3%	3.05 × 10^−70^	Extended Gly-X-Y-rich region; short cysteine-rich C-terminal region.
preCol-P (O16161)	TRINITY_DN376395_c3_g3_i5	905	51.3%	2.29 × 10^−134^	Central Gly-X-Y-rich region with compositionally distinct Ser/Ala/Gly/His-rich flanking regions; consistent with preCol-P-like organization.

The Mfp-2 and preCol searches also illustrate a common interpretive limitation of transcriptome-guided biomaterial discovery: recurring structural motifs such as EGF-like repeats or Gly-X-Y collagen repeats can occur in proteins with very different biological functions. Candidate assignment therefore requires evaluation of overall sequence context and architecture rather than reliance on isolated domains or repetitive motifs alone.

## 8. Conclusions

*Bathymodiolus azoricus* occupies an unusual position at the intersection of deep-sea adaptation, innate immunity, host–symbiont biology, bioadhesion, and biomineralization. The evidence reviewed here shows that its biomedical relevance is supported at several different levels. Direct experimental studies establish the species as a valuable system for investigating innate immune responses, host–microbe interactions, shell repair, hemocyte participation in biomineralization, and pressure-sensitive shell integrity. Targeted transcriptomic reanalyses further identify candidate molecular families of potential biomaterials interest, including an Mfp-2-like adhesive protein and preCol-D-, preCol-NG-, and preCol-P-like transcripts in foot tissue. These transcriptomic findings provide a basis for prioritizing molecules for future study, but do not by themselves establish protein production, post-translational modification, material performance, or biomedical function.

The central hypothesis advanced in this Review therefore remains deliberately testable rather than demonstrated: long-term exposure to the metal-rich hydrothermal environment may have influenced the chemistry and structural properties of *B. azoricus* adhesive and byssal systems. Established DOPA–metal coordination mechanisms in shallow-water mussels provide a mechanistic rationale for this possibility, but no direct comparative evidence currently shows enhanced Fe^3+^ binding, cross-linking, wet adhesion, self-healing, or mechanical performance in *B. azoricus*. Similarly, the distinctive environmental context of this species should not be taken as evidence of superior biomedical properties without direct functional comparison.

The next priorities are therefore clear: biochemical characterization of native or recombinant Mfp and preCol candidates; determination of DOPA content and other relevant post-translational modifications; formal domain annotation and protein-level validation of leading transcript-supported candidates; comparative Fe^3+^-binding, adhesion, and mechanical studies against shallow-water mytilids; and molecular characterization of the mantle through transcriptomic and shell-matrix proteomic approaches. These studies will determine whether *B. azoricus* represents primarily a deep-sea counterpart of established mussel biomaterial systems or whether hydrothermal adaptation has generated functionally distinct molecular designs that can be translated into engineered biomedical materials.

## Figures and Tables

**Table 2:** Relationship between three targets experimentally examined by RT-PCR/qPCR in the original 2010 study [10] and transcriptomic candidates recovered in the present 2010 and 2024 reanalyses.

Gene	Originally Published and RT-PCR/qPCR Examined (2010)	2010 Reanalysis	2024 Reanalysis	Interpretation
Integrin	Yes; included in the original experimental panel	6 candidate proteins recovered	32 transcripts recovered	Consistent molecular-family recovery across the original study and both reanalyses; current transcript searches do not constitute new experimental validation.
EGF	Yes; original contig c3243 in the signaling panel	29 EGF-domain-containing proteins in a broad domain sweep	3 ligand-focused EGF-domain candidates	EGF-associated candidates recovered in both reanalyses, but the two searches used different domain specificity.
Aggrecan/proteoglycan	Aggrecan contig lrc83347 was RT-PCR-confirmed in the original study	Aggrecan not recovered; only weak proteoglycan-related hits	Core proteoglycan candidates recovered, including a basement-membrane heparan-sulfate-proteoglycan-like sequence	The 2024 proteoglycan recovery does not independently confirm aggrecan specifically; the original aggrecan finding remains supported by the published RT-PCR result.

## Data Availability

The 454 gill-tissue sequencing reads originally reported in [10] are publicly available through the NCBI Sequence Read Archive under accession SRR067874. The original DeepSeaVent web portal is no longer accessible. The present Review therefore uses an independent reprocessing and annotation of the archived reads as described in Box 1. The resulting reassembly, annotation outputs, and queryable SQLite database supporting the targeted analyses reported here are available from the corresponding author upon reasonable request. The independently generated 2024 gill transcriptome is publicly available through the NCBI Sequence Read Archive under BioProject PRJNA1508204, SRA accession SRR40024054, and BioSample SAMN62218460. The foot-tissue sequencing data are likewise publicly available under BioProject PRJNA1508337, SRA accession SRR40036597, and BioSample SAMN62230578. Analysis of the foot transcriptome followed the workflow described in Box 2, including the targeted reference-guided searches used to identify Mfp- and preCol-like candidates.

## Abbreviations

The following abbreviations are used in this manuscript:

AIF1	Allograft Inflammatory Factor 1
ALDH	Aldehyde Dehydrogenase
BLAST	Basic Local Alignment Search Tool
CA	Carbonic Anhydrase
CBB	Calvin–Benson–Bassham cycle
DIAMOND	DIAMOND protein sequence aligner
DOPA	3,4-Dihydroxyphenyl-L-alanine
EGF	Epidermal Growth Factor
EPAS1	Endothelial PAS Domain Protein 1
ERK	Extracellular Signal-Regulated Kinase
FGF	Fibroblast Growth Factor
FGFR4	Fibroblast Growth Factor Receptor 4
HGF	Hepatocyte Growth Factor
HSP70	Heat Shock Protein 70
IGF	Insulin-like Growth Factor
JNK	c-Jun N-terminal Kinase
LITAF	Lipopolysaccharide-Induced TNF Factor
MAP	Mussel Adhesive Protein
MAPK	Mitogen-Activated Protein Kinase
MAR	Mid-Atlantic Ridge
MeDH	Methanol Dehydrogenase
Mfp	Mussel Foot Protein
MIRA	MIRA sequence assembler
MMO	Methane Monooxygenase
MMP	Matrix Metalloproteinase
NCBI	National Center for Biotechnology Information
ORF	Open Reading Frame
preCol	Mussel byssal pre-collagen protein
RGD	Arginine–Glycine–Aspartate peptide motif
RT-PCR	Reverse Transcription Polymerase Chain Reaction
RTK	Receptor Tyrosine Kinase
SEM	Scanning Electron Microscopy
SOXB	Sulfur Oxidation Gene B
SRA	Sequence Read Archive
SRCR	Scavenger Receptor Cysteine-Rich domain
TGF-β	Transforming Growth Factor Beta
TIMP	Tissue Inhibitor of Metalloproteinases
TLR2	Toll-Like Receptor 2
TNF	Tumor Necrosis Factor
VEGF	Vascular Endothelial Growth Factor
WGA	Wheat Germ Agglutinin
